# Transgenic Expression of a Single Transcription Factor Pdx1 Induces Transdifferentiation of Pancreatic Acinar Cells to Endocrine Cells in Adult Mice

**DOI:** 10.1371/journal.pone.0161190

**Published:** 2016-08-15

**Authors:** Satsuki Miyazaki, Fumi Tashiro, Jun-ichi Miyazaki

**Affiliations:** Division of Stem Cell Regulation Research, Osaka University Graduate School of Medicine, Suita, Osaka, 565-0871, Japan; Centro Nacional de Investigaciones Oncologicas, SPAIN

## Abstract

A promising approach to new diabetes therapies is to generate β cells from other differentiated pancreatic cells *in vivo*. Because the acinar cells represent the most abundant cell type in the pancreas, an attractive possibility is to reprogram acinar cells into β cells. The transcription factor Pdx1 (Pancreas/duodenum homeobox protein 1) is essential for pancreatic development and cell lineage determination. Our objective is to examine whether exogenous expression of Pdx1 in acinar cells of adult mice might induce reprogramming of acinar cells into β cells. We established a transgenic mouse line in which Pdx1 and EGFP (enhanced green fluorescent protein) could be inducibly expressed in the acinar cells. After induction of Pdx1, we followed the acinar cells for their expression of exocrine and endocrine markers using cell-lineage tracing with EGFP. The acinar cell-specific expression of Pdx1 in adult mice reprogrammed the acinar cells as endocrine precursor cells, which migrated into the pancreatic islets and differentiated into insulin-, somatostatin-, or PP (pancreatic polypeptide)-producing endocrine cells, but not into glucagon-producing cells. When the mice undergoing such pancreatic reprogramming were treated with streptozotocin (STZ), the newly generated insulin-producing cells were able to ameliorate STZ-induced diabetes. This paradigm of *in vivo* reprogramming indicates that acinar cells hold promise as a source for new islet cells in regenerative therapies for diabetes.

## Introduction

In the healthy pancreas, the β-cell mass changes throughout life in response to insulin demand. It increases both through an increase in the volume of existing β cells and through their proliferation [1–3]. By tracing the lineage of genetically marked β cells in mice, Dor et al. [4] showed that following birth or a 70% pancreatectomy, new β cells are mostly formed by self-replication. However, they can also be generated by β-cell neogenesis. In animal models for β-cell regeneration, induced by partial pancreatectomy [5], cellophane wrapping [6], duct ligation [7], or interferon-γ overexpression [8], new β cells appear to be generated. Although the mechanism for the β-cell regeneration has not been clarified, transdifferentiation into β cells from duct cells [5–8], acinar cells [9,10], centroacinar cells [11], and other endocrine cells such as α cells and δ cells [12–15] has been reported. In particular, in studies on acinar-to-β-cell transdifferentiation *in vivo*, cells coexpressing insulin and amylase have been reported [9,16,17]. In addition, primary rat acinar cells spontaneously dedifferentiate *in vitro*, thereby losing acinar characteristics, such as digestive-enzyme expression, and gaining embryonic and ductal characteristics, such as expression of the transcription factor Pdx1 (Pancreas/duodenum homeobox protein 1) and certain cytokeratins [18,19]. This process is called “acinar-to-ductal transdifferentiation”. Similar phenomena have been reported in mice [20] and humans [21], and these transdifferentiated duct-like cells can be redirected into endocrine lineages [22]. Insulin-secreting cells were also generated from murine acinar cells by suspension culture [23]. Thus, differentiated acinar cells seem to retain considerable plasticity, which may permit them to be reprogrammed into β cells.

On the other hand, a study using *in vivo* cell-lineage tracing showed that acinar cells contribute only to acinar cell regeneration, not to β-cell regeneration, in models of pancreatitis caused by partial pancreatectomy, cerulein injection, or pancreatic duct ligation [24]. Strobel et al. [25] also used *in vivo* genetic cell-lineage tracing to examine whether the transdifferentiation of acinar cells plays a role in regeneration and metaplasia in pancreatitis. Their results showed that acinar cells are regenerated only from preexisting acinar cells, and that acinar-to-ductal transdifferentiation occurs in the pancreas of adult mice, but makes only small contributions to metaplastic lesions. These results suggest that mature acinar cells have only a limited plasticity for transdifferentiation. Furthermore, Xiao et al. [26] recently used a novel mouse model for detecting new β cells derived from non-β cells and showed that β-cell neogenesis may not make major contributions to the postnatal β-cell pool in most physiological and pathological conditions. Similar results were also reported by Rankin et al. [27]. Thus, there is a major discrepancy in regard to the plasticity of acinar cells.

Another strategy employed to induce transdifferentiation of pancreatic cells in mice is to exogenously express key developmental transcription factor(s). Pdx1, a homeodomain-containing transcription factor, is an essential regulator of pancreatic endocrine development and adult islet β-cell function [28]. Ablating Pdx1 by gene targeting blocks pancreatic development at an early stage, showing that embryonic Pdx1-expressing pancreatic progenitors give rise to the entire pancreas, i.e., the duct, exocrine, and endocrine tissues [29,30]. Pdx1 is upregulated in the regenerating pancreas [31,32] and in cultured acinar cells during their dedifferentiation [17], suggesting that transcriptional regulation by Pdx1 is essential, not only for pancreatic development, but also for pancreatic regeneration. In fact, we previously showed that adenovirus vector-mediated expression of Pdx1 in the exocrine pancreas induces tubular complex formation and β-cell neogenesis [33]. Miyatsuka et al. [34] showed that the pancreatic acinar-cell-specific overexpression of Pdx1 during the fetal-to-neonatal period causes acinar-to-ductal transdifferentiation. We also showed that Pdx1 expression facilitates tubular complex formation through acinar-to-ductal metaplasia induced by delivery of adenovirus vector expressing Isl1, a proendocrine transcription factor, into the exocrine pancreas of adult mice [35]. Heller et al. [36] generated transgenic (Tg) mice in which Pdx1 was expressed in the exocrine pancreas under the elastase-1 promoter. These mice showed marked dysmorphogenesis of the exocrine pancreas, accompanied by increased rates of both the replication and apoptosis of acinar cells. Amylase/insulin double-positive cells were observed in the pancreas of the Tg mice on embryonic day 18, suggesting that transdifferentiation could be taking place. In addition, more single insulin-positive cells were found in the exocrine pancreas of the Tg mice than in that of normal mice at 4 weeks of age, suggesting there was increased β-cell neogenesis in the Tg mice. Yang et al. [37] reported that exogenous Pdx1 expression in Neurogenin 3 (Ngn3)-expressing endocrine progenitor cells of embryos caused a minor increase of β-cell numbers accompanied by reduced α-cell numbers during the embryonic period and an almost complete α-to-β cell conversion at postnatal stages through glucagon/insulin double-positive cells. These results indicate that transgenic expression of Pdx1 enhances the plasticity of pancreatic acinar and other cells, and induces their transdifferentiation, leading to β-cell neogenesis. However, the effects of long-term expression of Pdx1 at adult age on the differentiation status or the plasticity of acinar cells have not been reported.

We previously established RTF-Pdx1-EGFP mice, which inducibly express Pdx1/EGFP (enhanced green fluorescent protein). When Pdx1 expression was induced for 3 weeks in adult RTF-Pdx1-EGFP mice, however, we could not observe any pathological changes in the pancreas [35]. In the present study, we used a Tg mouse line that has acinar-cell-specific inducible Pdx1/EGFP double expression and performed cell-lineage tracing to examine the effects of long-term expression of Pdx1 on the plasticity of acinar cells in adult mice.

## Materials and Methods

### Pdx1-expressing mice

We previously established an RTFN-Pdx1-EGFP mouse line, in which tTA is expressed upon Cre-mediated deletion of the loxP-flanked neo^r^ gene under control of the *ROSA26* locus promoter [35,38,39]. This line was maintained by crosses with C57BL/6J mice. For acinar-cell-specific expression of Pdx1 and EGFP, the RTFN-Pdx1-EGFP mice were mated with Elastase-Cre Tg mice, in which Cre is expressed specifically in acinar cells [34,40]. The resulting double heterozygous progeny, designated ERTF-Pdx1-EGFP, were maintained under a continuous administration of Dox (doxycycline hydrochloride; Sigma-Aldrich, St. Louis, MO) in the drinking water at 0.05 mg/ml in light-protected bottles, to suppress Pdx1/EGFP expression. Four- to five-week-old ERTF-Pdx1-EGFP mice and their littermates were used in this study. Mice were housed and maintained in a controlled environment according to the institutional guidelines. Our animal institute has obtained the Animal Welfare Assurance (#A5950-01) from the Office of Laboratory Animal Welfare (OLAW) of National Institutes of Health, USA. All experiments involving animals were carried out in accordance with the institutional guidelines under the protocols (#21–089 and #26–066), which were approved by the Animal Care and Use Committee of the Osaka University Graduate School of Medicine. The physical condition of the experimental animals was monitored every two or three days. Because mice treated with streptozotocin (STZ) became diabetic, we monitored their body weight (BW) twice a week. We regarded loss of > 20% of BW compared to the pre-study BW as the clinical sign for humane endpoints, but STZ-treated mice did not lose more than 10% of BW. None of the animals became severely ill or died prior to the experimental endpoint. Mice were euthanized with an intraperitoneal injection of pentobarbital sodium at 180 mg/kg BW.

### Immunofluorescence analysis

Pancreatic tissue was fixed in 4% paraformaldehyde overnight and processed for paraffin embedding. Sections of paraffin-embedded pancreatic tissue (3~5-μm-thick) were deparaffinized, dehydrated, and incubated with 3% normal goat serum in phosphate buffered saline (PBS) containing 10% Blocking One (Nacalai Tesque, Kyoto, Japan) for 60 min at room temperature. The sections were then incubated with the first antibody at 4°C overnight and with a fluorescein-conjugated second antibody for 60 min at room temperature. After each antibody incubation, the sections were washed in PBS for 5 min with three changes. The first antibodies were guinea pig anti-insulin (Dako, Carpinteria, CA), rabbit anti-amylase (Sigma-Aldrich, St. Louis, MO), rat anti-cytokeratin 19 (CK19) monoclonal (TROMA-III; Developmental Studies Hybridoma Bank, Iowa City, IA), mouse anti-HNF1β (BD Pharmingen, San Diego, CA), mouse anti-Nkx6.1 monoclonal (N55A10; Developmental Studies Hybridoma Bank, Iowa City, IA), goat anti-E-cadherin (R&D Systems, Minneapolis, MN), rabbit and chicken anti-GFP (Abcam, Cambridge, MA), rabbit anti-Pdx1 (Trans Genic Inc., Kumamoto, Japan), rabbit anti-MafB (Bethyl, Montgomery, TX), rabbit anti-somatostatin (Progen Biotechnik, Heidelberg, Germany and DiaSorin, Saluggia, Italy), guinea pig anti-glucagon, guinea pig anti-PP (Linco, St. Charles, MO), guinea pig anti-C-peptide (Takara, Shiga, Japan), and biotinylated rat anti-mouse CD31 (PECAM-1) (AbD Serotec, Oxford, UK) antibodies. The second antibodies were Alexa Fluor 488- or 568-conjugated anti-rabbit IgG, Alexa Fluor 594-conjugated anti-rat IgG, Alexa Fluor 488-conjugated anti-mouse IgG_1_, Alexa Fluor 488-conjugated anti-chicken IgG, and Alexa Fluor 594-conjugated anti-guinea pig IgG (Molecular Probes, Eugene, OR). To detect HNF1β, E-cadherin, and PECAM-1, we used the TSA system (Perkin Elmer, Norwalk, CT) in combination with HRP (horseradish peroxidase)-conjugated goat anti-mouse IgG_1_, HRP-conjugated donkey anti-goat IgG, and streptavidin-Alexa 647 (SA-647) for signal amplification, according to the manufacturer’s instructions. Nuclei were stained with DAPI (4’, 6-diamidino-2-phenylindole). The sections were observed by immunofluorescence microscopy (Keyence, Osaka, Japan; Olympus, Tokyo, Japan).

For the determination of BrdU (5-bromo-2'-deoxyuridine) incorporation, mice were administered with BrdU-labeling reagent 24 h before being killed 6 to 7 weeks after Dox withdrawal. Immunohistochemical staining for BrdU was performed by using rat anti-BrdU antibody (Abcam). The sections were incubated in 1 M HCl for 20 min at room tempreture prior to the blocking step.

### Evaluation of histological changes

The whole pancreas was excised, coiled up (so that both the proximal and distal parts of the pancreas could be observed on the same section), and placed onto a piece of waxed paper. Three consecutive paraffin sections from each pancreas, with the largest possible cross-sections chosen, were stained with hematoxylin-eosin or with the anti-GFP antibody and anti-insulin antibodies. Each section was examined by immunofluorescence microscopy and digitally scanned. The image files of the islets stained with these antibodies were analyzed, and the number of islet cells stained with the anti-GFP antibody, the anti-insulin antibody, and both antibodies was counted for each islet.

### Streptozotocin (STZ) treatment

STZ (Sigma, St. Louis, MO) was prepared in citrate buffer (pH 4.5) and a one- or two-time dose of 160 mg/kg was delivered to mice by intraperitoneal injection. The ERTF-Pdx1-EGFP mice were treated with STZ while under Dox administration (Dox (+) STZ (+)) or 4 weeks after Dox withdrawal (Dox (-) STZ (+)). Mice were given food *ad libitum*. At intervals of 3–10 days, blood samples were obtained from the tail vein, and the glucose levels were measured using a blood glucose test meter (Sanwa Kagaku, Nagoya, Japan).

### Intraperitoneal glucose tolerance test (ipGTT)

The ipGTT was performed on four groups of mice untreated or treated with STZ: wild-type mice (STZ (-)), ERTF-Pdx1-EGFP mice (Dox (-) STZ (-)), ERTF-Pdx1-EGFP mice (Dox (-) STZ (+)), and ERTF-Pdx1-EGFP mice (Dox (+) STZ (+)). Six weeks after STZ treatment, the mice were fasted overnight for 16 h and then given an intraperitoneal glucose injection (2 g/kg body weight). Blood samples were obtained from the tail vein, and the blood glucose and serum insulin concentrations were measured using a blood glucose test meter (Sanwa Kagaku) and an enzyme-linked immunosorbent assay (ELISA) kit for mouse insulin (Mercodia, Uppsala, Sweden), respectively.

### Statistical analysis

The statistical analysis was performed by Student’s *t-*test or by one-way ANOVA followed by Tukey’s post-hoc test for comparing more than two groups. The *P*-values for significance were set to 0.05. Data are presented as means ± SE.

## Results

### Acinar-cell-specific inducible expression of Pdx1

**Fig 1:**
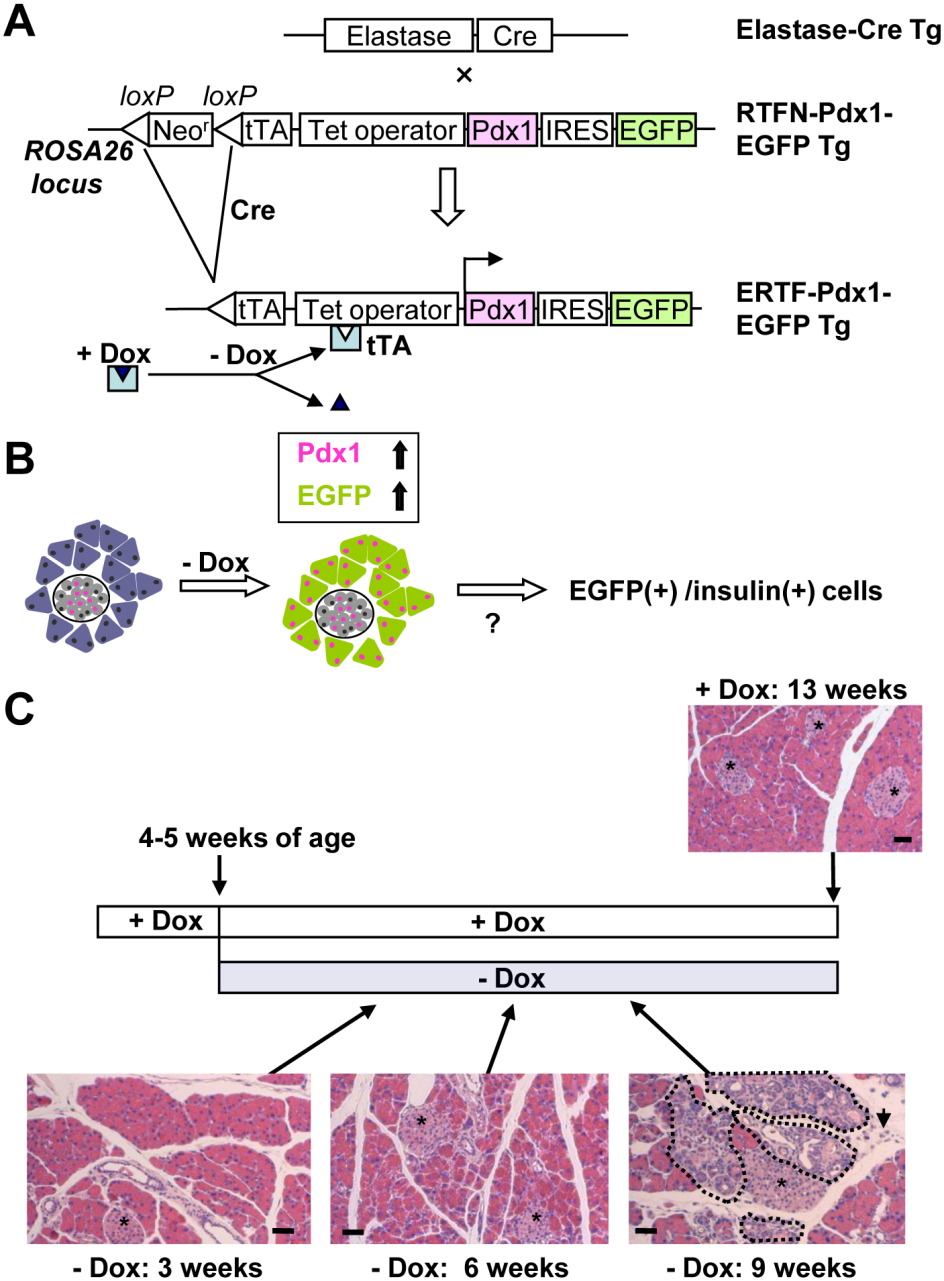
Cre-mediated lineage tracing approach. (A) Elastase-Cre Tg mice, in which the elastase promoter drives the expression of Cre in pancreatic acinar cells, were crossed with RTFN-Pdx1-EGFP mice. The double heterozygous ERTF-Pdx1-EGFP mice lost the *loxP*-flanked neo^r^ gene in the acinar cells. Pdx1 and EGFP were reversibly induced in these cells by withdrawing Dox, which had been added to the drinking water to suppress their expression. (B) Schematic illustration of our hypothesis that if acinar-to-β-cell transdifferentiation/reprogramming could be induced by the sustained high expression of Pdx1, we might find EGFP-positive insulin-producing cells in the exocrine pancreas and/or in islets in the ERTF-Pdx1-EGFP mice. (C) Time course and histology. Dox was withdrawn from the drinking water of 4-5-week-old ERTF-Pdx1-EGFP mice, and histological changes of the pancreas were examined 3–9 weeks later (hematoxylin and eosin staining). Lower right panel: The areas surrounded by dotted lines represent tubular complexes. Arrow shows an increase of mesenchymal cells and connective tissue. Asterisks indicate islets. Bars = 50 μm.

To study the effect of Pdx1 expression on acinar-cell plasticity in adults, we crossed two previously established Tg mouse lines, RTFN-Pdx1-EGFP [35,38,39] and Elastase-Cre [34,40], to make ERTF-Pdx1-EGFP mice (see Materials and Methods). These mice had lost the *loxP*-flanked neomycin-resistance gene and expressed the tetracycline-repressible transactivator (tTA) protein specifically in the pancreatic acinar cells. Thus, in these mice, Pdx1 and EGFP were expressed only in acinar cells, and their expression could be repressed by the tetracycline analogue, Dox (Fig 1A). We took advantage of the EGFP labeling to determine if the overexpression of Pdx1 affected the differentiation status of the acinar cells (Fig 1B). When Dox was removed from the drinking water from the time of conception, the ERTF-Pdx1-EGFP progeny mice showed severe dysmorphogenesis of the exocrine pancreas at 2 weeks of age (S1 Fig), quite similar to that observed in the mice expressing Pdx1 specifically in the pancreas from the prenatal period [34]. Since the life span of the ERTF-Pdx1-EGFP mice was 2–3 weeks, we could not examine the histological changes of the adult mouse pancreas.

In the present study, we induced Pdx1 expression in the adult ERTF-Pdx1-EGFP mice by Dox withdrawal from the drinking water. It should be noted that in the study involving Tg mouse using the Elastase promoter, leakage of the transgene expession was sometimes observed in islets [25]. To assess the extent of spontaneous activation of the Elastase-Cre transgene in islet cells, we made a control mouse line carrying the Tet-off knock-in cassette of RTFN-EGFP, lacking the Pdx1 cDNA. This line was crossed with Elastase-Cre Tg mouse line to produce ERTF-EGFP Tg mice, which were maintained without Dox for 3, 6, and 9 weeks from 4 to 5 weeks of age. EGFP expression gradually increased after Dox withdrawal (S2A–S2C Fig). There were no apparent histological changes in the pancreas sections from these mice. Nine weeks after Dox withdrawal, almost all acinar cells were EGFP-positive (S2D Fig), but EGFP-positive cells were hardly observed in the ducts or islets (S2E–S2J Fig). We examined pancreas sections from four ERTF-EGFP Tg mice 9 or 16 weeks after Dox withdrawal, but EGFP/insulin double-positive cells were not found. Thus, the spontaneous leak of the Cre expession was considered negligible in the Elastase-Cre mouse line we used in this study.

**Fig 2:**
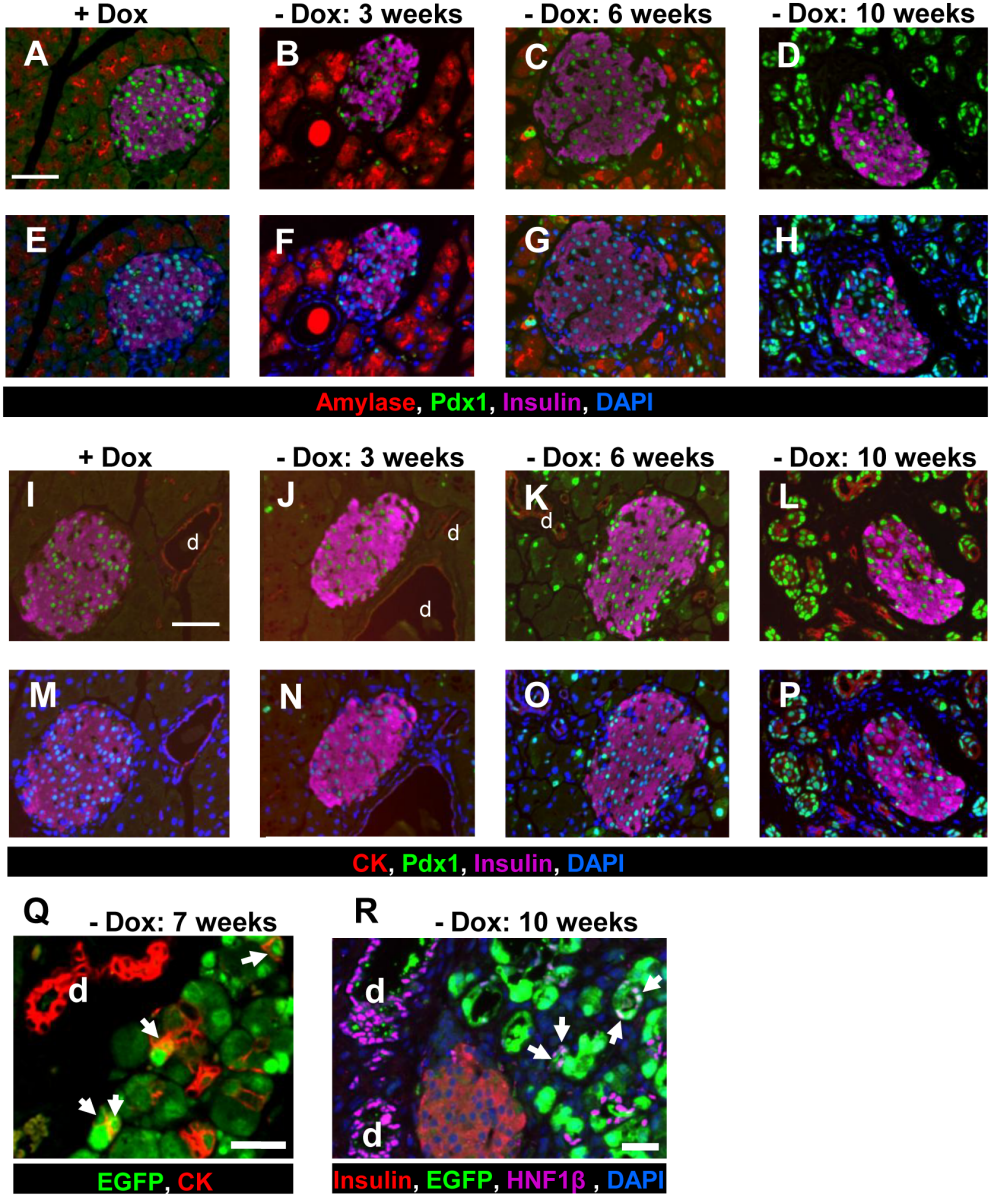
Immunohistochemical analysis of the pancreas of ERTF-Pdx1-EGFP mice. (A-H) Pancreas sections from ERTF-Pdx1-EGFP mice were stained for amylase (red), Pdx1 (green), insulin (purple), and DNA (blue). Strong ectopic expression of Pdx1 in the acinar cells was induced when Dox was withheld from the drinking water for more than 6 weeks. Only the islets were positive for Pdx1 in Dox (+) control pancreas sections as shown in (A) and (E). Bar = 50 μm. (I-P) Pancreas sections from ERTF-Pdx1-EGFP mice were stained for CK19 (red), Pdx1 (green), insulin (purple), and DNA (blue). Strong ectopic expression of Pdx1 in the CK19-positive duct cells was observed when Dox was withheld from the drinking water for more than 10 weeks. “d” represents duct cells. Bar = 50 μm. (Q) Pancreas sections from ERTF-Pdx1-EGFP mice 7 weeks after Dox withdrawal were stained for CK19 (red), and EGFP (green). Arrows indicate EGFP/CK19 double-positive cells. “d” represents duct cells. Bar = 25 μm. (R) Pancreas sections from ERTF-Pdx1-EGFP mice without Dox treatment for 10 weeks were stained for insulin (red), EGFP (green), HNF1β (purple), and DNA (blue). Arrows indicate EGFP/HNF1β double-positive cells, which were detected from around 6 weeks after Dox withdrawal and were probably derived from acinar cells, suggesting that acinar-to-ductal transdifferentiation occurred. Arrows indicate double-positive cells. “d” represents duct cells. Bar = 25 μm.

Because the maturation of pancreatic islets is thought to end at around 4 weeks of age [41,42], Dox was withdrawn from the drinking water when the ERTF-Pdx1-EGFP mice were 4–5 weeks old. To evaluate the histological changes, we examined the pancreas from the ERTF-Pdx1-EGFP mice 3, 6, and 9 weeks after Dox withdrawal. Three weeks after Dox withdrawal, a small number of Pdx1-expressing acinar cells were observed (Fig 2B, 2F, 2J and 2N). No obvious histological differences were seen between the experimental mice (Fig 1C:—Dox 3 weeks) and the control Dox (+) ERTF-Pdx1-EGFP mice, which were given Dox continuously (Fig 1C: + Dox 13 weeks). Six weeks after Dox withdrawal, strong ectopic expression of Pdx1 was extensively seen in acinar cells (Fig 2C, 2G, 2K and 2O). From 8 weeks after Dox withdrawal, we noted apparent histological changes including tubular complex formation and an increase of mesenchymal cells and connective tissue (Fig 1C:—Dox 9 weeks). Tubular complexes were found throughout the pancreas as shown in the areas surrounded by dotted lines in Fig 1C (- Dox 9 weeks). Pdx1 expression was clearly observed in most of acinar cells, especially those undergoing morphological change (Fig 2D, 2H, 2L and 2P). At this stage, the cytoplasmic volume of acinar-derived cells appeared to be reduced and their expression of amylase was scarcely detectable. We speculated that these reduced-cytoplasm cells were undergoing acinar-to-ductal transdifferentiation, considering a dramatic increase of Pdx1/CK19 (a duct cell marker) double-positive cells (Fig 2L and 2P). To verify this interpretation, we immunostained pancreas sections from ERTF-Pdx1-EGFP mice for EGFP and CK19 or another duct cell marker, HNF1β. In fact, cells co-expressing EGFP and a duct cell marker were seen from 7 weeks after Dox withdrawal (Fig 2Q and 2R).

### New insulin-positive cells derived from exocrine cells

**Fig 3:**
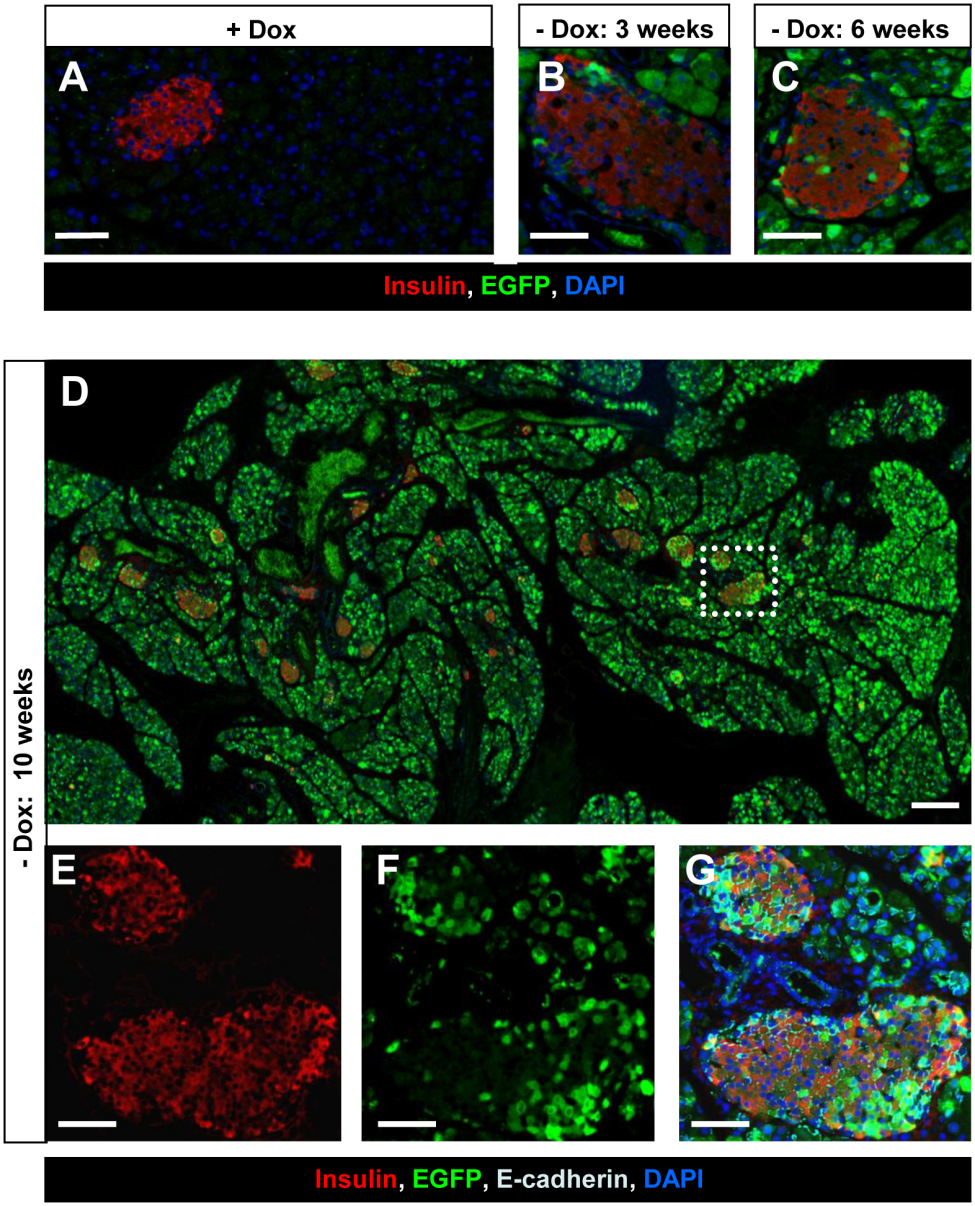
Immunohistochemical analysis of the acinar-to-β-cell transdifferentiation/reprogramming. **(A-C)** Sections from the pancreas of ERTF-Pdx1-EGFP mice 3, 6, and 10 weeks after Dox withdrawal were stained for insulin (red), EGFP (green), and DNA (blue). The presence of EGFP-positive cells expressing insulin suggests the occurrence of acinar-to-β-cell transdifferentiation/reprogramming. No EGFP-positive cells were seen when Dox treatment was continued (A). Bars = 50 μm. (D) Pancreas sections were obtained from ERTF-Pdx1-EGFP mice 10 weeks after Dox withdrawal and stained for insulin (red), EGFP (green), and DNA (blue). Bar = 200 μm. (E-G) Magnified views of the dotted line-box in (D) are shown. Staining of E-cadherin (light blue) was included in (G). Bars = 50 μm. A substantial number of EGFP-positive islet cells were insulin-positive, and these double-positive cells were evenly distributed in the islets. These changes in islets were seen all over the pancreas (D).

We further followed the fate of the Pdx1-expressing acinar cells in the pancreas (Fig 1B). To determine whether the Pdx1 expression induced new insulin-positive cells, we stained pancreas sections for EGFP and insulin by double immunofluorescence. Three weeks after Dox withdrawal, EGFP-positive cells were seen in the islet periphery, but most of them were insulin-negative (Fig 3B). Six weeks after Dox withdrawal, an increase in peripheral EGFP-positive cells co-expressing insulin was seen (Fig 3C). Ten weeks after Dox withdrawal, a substantial number of EGFP-positive cells within islets were insulin-positive (380 of 431 EGFP-positive islet cells), and these double-positive cells were evenly distributed in the islets (Fig 3E–3G). These changes were seen all over the pancreas (Fig 3D). Similar results were obtained by double staining for C-peptide and EGFP (S3A–S3D Fig). On the other hand, no EGFP-positive cells were observed in the pancreas of the Dox (+) control mice (Fig 3A; S3E Fig).

**Fig 4:**
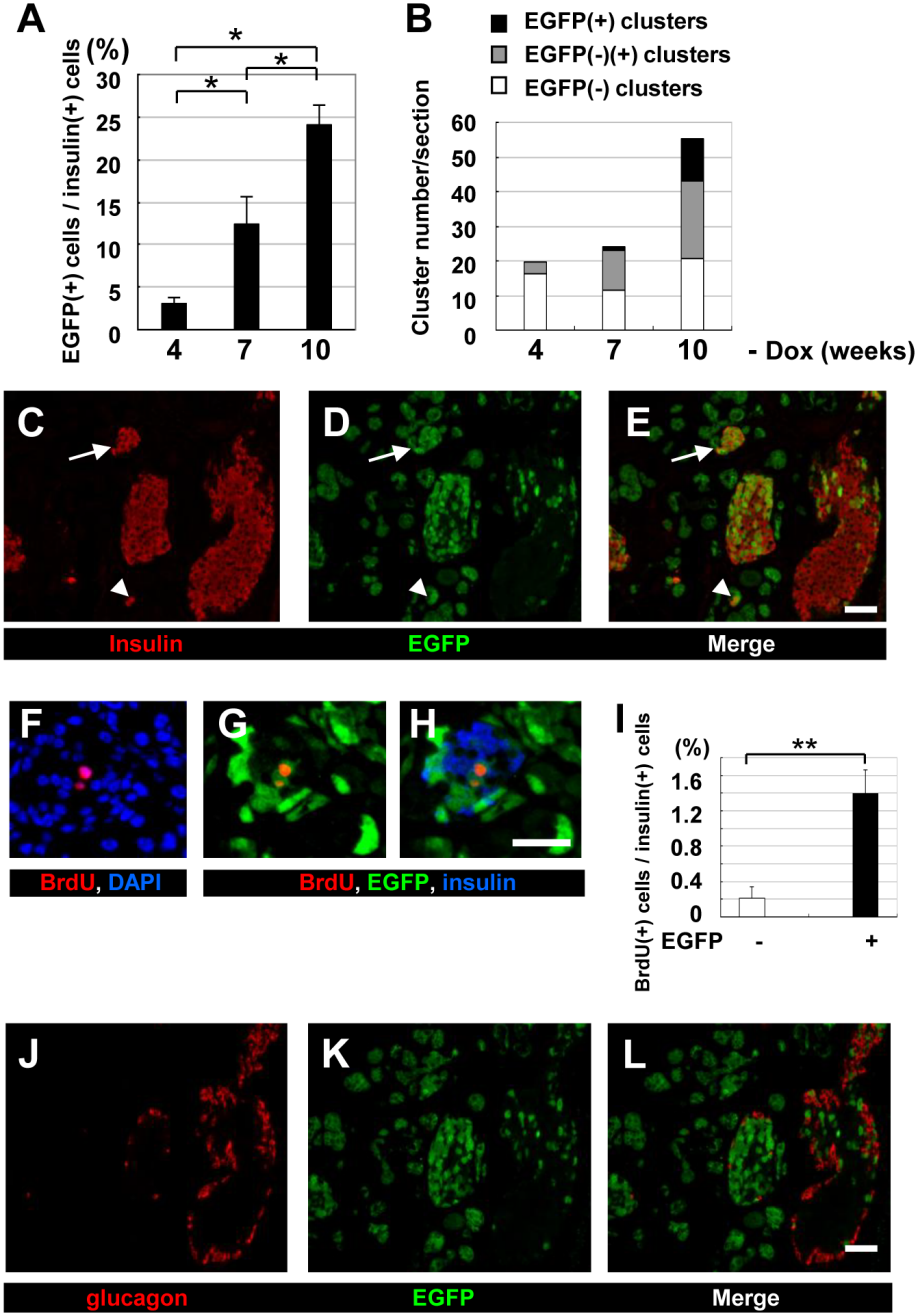
Immunohistochemical analysis of the pancreas of ERTF-Pdx1-EGFP mice maintained without Dox. Sections from the pancreas of ERTF-Pdx1-EGFP mice 4, 7, and 10 weeks after Dox withdrawal (*n* = 3, 3, and 4, respectively) were stained for insulin, EGFP, and DNA. (A) The percentage of EGFP-positive cells among insulin-positive islet cells was calculated for each mouse. The total number of insulin-positive cells examined was 4968, 6690, and 9555 at 4, 7, and 10 weeks after Dox withdrawal, respectively. Statistical analysis was performed by one-way ANOVA followed by Tukey’s post-hoc test. *P < 0.05. (B) EGFP expression in islet-like clusters containing fewer than 10 insulin-positive cells was examined for the pancreas of these mice. The percentage of islet-like clusters containing insulin-producing cells with only EGFP-negative (open bars), EGFP-positive and negative (grey bars), and only EGFP-positive (black bars) cells was determined. (C-E) Immunohistochemical analysis of the pancreas of ERTF-Pdx1-EGFP mice 10 weeks after Dox withdrawal. Sections were stained for insulin (red) and EGFP (green). Right panel (E) is a merged view of (C) and (D). Arrow shows an islet-like cluster in which all the insulin-positive cells were also EGFP-positive. Arrowhead shows EGFP-positive insulin-producing cells in an islet-like cluster. Bar = 50 μm. (F-I) Increased BrdU-positive cells in the islets of ERTF-Pdx1-EGFP mice 6 weeks after Dox withdrawal. Pancreas sections were stained for BrdU (red), DNA (blue), and EGFP (green). Merged image of (G) with insulin staining (blue) is shown in (H). Bar = 25 μm. The percentage of BrdU-positive cells among EGFP-negative and -positive insulin-positive islet cells was determined (I). In the islets, BrdU-positive cells were significantly more enriched in EGFP/insulin double-positive cells than in insulin-positive EGFP-negative cells. The total number of insulin-positive islet cells examined was 1514, 1762, 2233, and 3375 each from four ERTF-Pdx1-EGFP mice. **P < 0.01. (J-L) Pancreas sections from ERTF-Pdx1-EGFP mice 10 weeks after Dox withdrawal were stained for glucagon (red) and EGFP (green). Serial section to that used in (C-E) was used. Right panel (L) is a merged view of (J) and (K). No EGFP/glucagon double-positive cells were observed. Bar = 50 μm.

Next, the proportion of EGFP-positive cells in the islets was determined at various time points after Dox withdrawal. Four weeks after Dox withdrawal, only 3% of the insulin-positive islet cells were positive for EGFP (Fig 4A). The percentage of EGFP-positive cells among the insulin-positive islet cells increased over time, reaching approximately one fourth at 10 weeks after Dox withdrawal (Fig 4A). We next extended our analysis to small, scattered islet-like clusters consisting of less than ten β cells (Fig 4B). In the Dox (-) group, the percentage of islet-like clusters containing EGFP-positive cells increased over time, and 10 weeks after Dox withdrawal, small clusters containing only EGFP/insulin double-positive cells were often found (Fig 4C–4E, arrows). These clusters may have arisen by direct acinar-to-β-cell transdifferentiation.

The increase in EGFP/insulin double-positive islet cells might have been due to self-replication. We tested this possibility by BrdU-labeling 6 to 7 weeks after Dox withdrawal which is two to three weeks before histological changes became the most prominent. The percentage of BrdU(+) cells among the EGFP(+)/insulin(+) islet cells was much higher than that among the EGFP(-)/insulin(+) islet cells (Fig 4F–4I). Thus, EGFP/insulin double-positive islet cells were considered to be more proliferative than preexisting β cells.

### Reprogrammed acinar cells generate endocrine progenitors

To characterize the EGFP-positive islet cells, we examined their expression of hormones besides insulin, including glucagon, somatostatin, and pancreatic polypeptide (PP) by immunohistochemistry. Three to ten weeks after Pdx1 induction, we found EGFP-positive islet cells expressing PP (S4A–S4D Fig) or somatostatin (S4E–S4I Fig). On the other hand, we could not find any EGFP-positive islet cells expressing glucagon (Fig 4J–4L). We also looked for EGFP-positive islet cells expressing amylase or CK19 in pancreas sections 3 to 10 weeks after Dox withdrawal, but did not find any EGFP-positive islet cells that co-expressed these markers (Fig 2).

**Fig 5:**
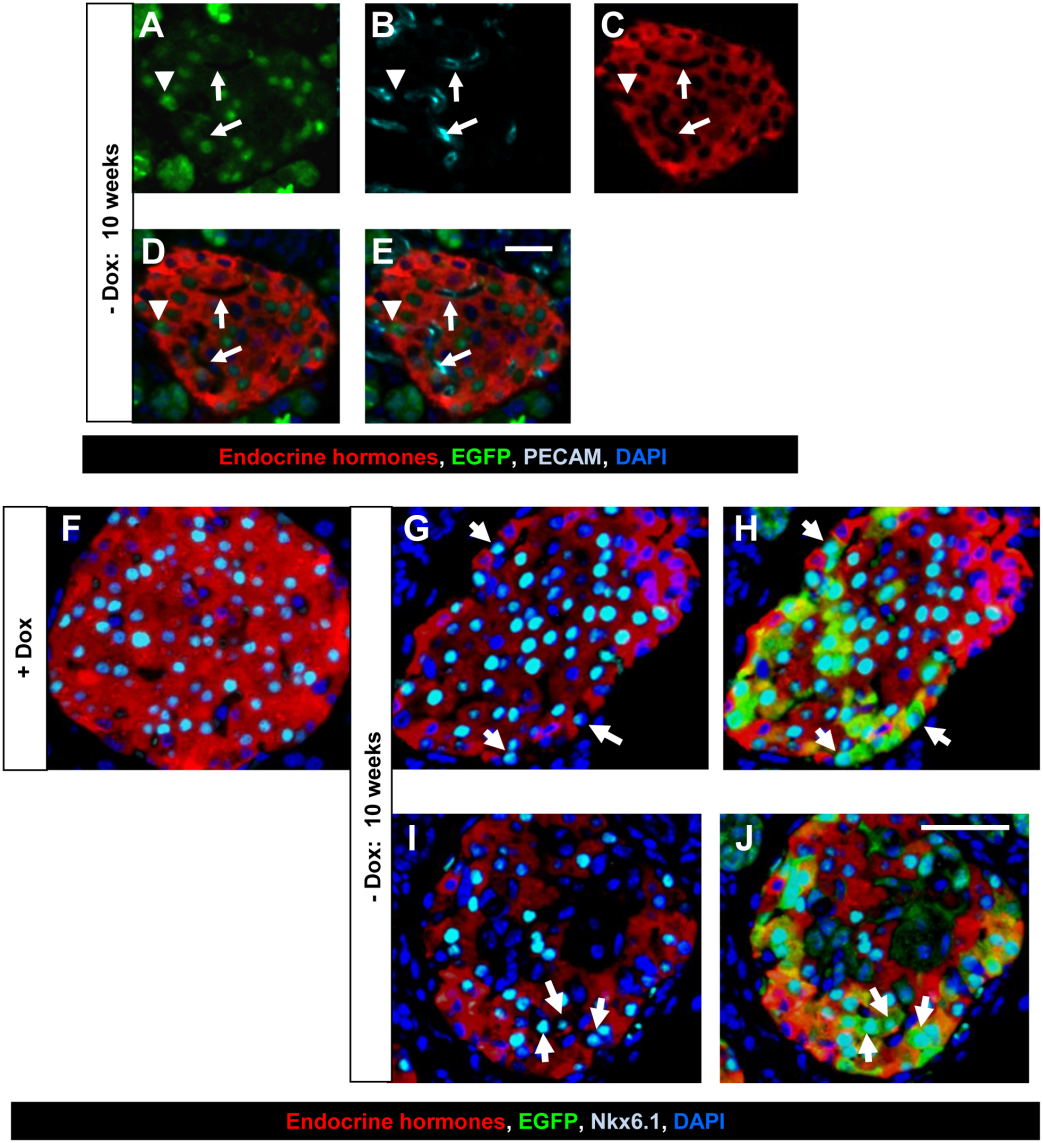
Detection of endocrine hormone-negative EGFP-positive islet cells. (A-E) PECAM-1 staining of endocrine hormone-negative EGFP-positive islet cells. Pancreas sections from ERTF-Pdx1-EGFP mice 10 weeks after Dox withdrawal were stained with the anti-GFP antibody (green), anti-PECAM-1 antibody (light blue), a mixture of antibodies against the endocrine hormones insulin, glucagon, PP, and somatostatin (red), and DAPI (blue). Single staining views and merged views are shown. Arrowhead indicates an endocrine hormone-negative EGFP-positive islet cell, which was also negative for PECAM-1, suggesting that it was not from the endothelial lineage. Arrows indicate endocrine hormone-negative EGFP-negative islet cells. These cells were positive for PECAM-1, suggesting that they were from the endothelial lineage. Bar = 50 μm. (F-J) Pancreas sections from ERTF-Pdx1-EGFP mice with Dox (F) and without Dox for 10 weeks (G-J) were stained with the anti-GFP antibody (green) (H, J), anti-Nkx6.1 antibody (light blue), a mixture of antibodies against the endocrine hormones insulin, glucagon, PP, and somatostatin (red), and DAPI (blue). Arrows indicate endocrine hormone-negative EGFP-positive Nkx6.1 positive islet cells. No endocrine hormone-negative Nkx6.1 positive islet cells were seen when Dox treatment was continued (F). Bar = 50 μm.

We speculated that the overexpressed Pdx1 induced acinar cells to dedifferentiate into immature, pancreatic stem/progenitor-like cells. These stem-like cells then migrated into the islets, where they redifferentiated into pancreatic endocrine cells, such as β cells, δ cells (which express somatostatin), and PP-producing cells. If this were the case, we should find EGFP-positive cells in islets that had not yet begun expressing hormones. To test this hypothesis, we performed immunofluorescence analyses using an anti-GFP antibody and a mixture of antibodies against the endocrine hormones insulin, glucagon, PP, and somatostatin. Three to ten weeks after Dox withdrawal, approximately 7 to 14% of the EGFP-positive islet cells were indeed negative for these hormones. To rule out the possibility that these endocrine-hormone-negative EGFP-positive islet cells were endothelial cells, we performed additional immunofluorescence analyses using an anti-PECAM-1 (platelet endothelial cell adhesion molecule-1) antibody (Fig 5A–5E). The results showed that these cells were negative for PECAM-1, and therefore not endothelial cells. To further characterize these endocrine-hormone-negative EGFP-positive islet cells, we performed immunofluorescence analyses using antibodies against transcription factors, MafA, MafB, Pax6, Ngn3, and Nkx6.1, which are specifically expressed in endocrine cells and/or their precursors. The results showed that these endocrine hormone-negative EGFP-positive islet cells were negative for MafA, MafB, Pax6, and Ngn3 (not shown), but some of them were positive for Nkx6.1 (Fig 5G–5J). Nkx6.1 is expressed in islet precursors and adult islet cells (Fig 5F) [43,44]. Therefore, endocrine hormone-negative EGFP-positive islet cells that expressed Nkx6.1 were likely to be precursors for β cells.

In these analyses, we found MafB-positive cells arose among the EGFP-positive acinar-derived cells of ERTF-Pdx1-EGFP mice 10 weeks after Dox withdrawal (S5 Fig). These MafB-positive cells comprised approximately 10% of EGFP-positive cells and were negative for endocrine hormones, amylase, and Hnf1β (S5D–S5Q Fig). MafB is required for β cell development and the switch from MafB to MafA expression is essential for functional maturation of β cells [45]. Therefore, these MafB/EGFP double-positive cells were likely to be precursors for EGFP/insulin double-positive cells in islets and/or islet-like clusters and their occurrence supports the view that Pdx1 expression in acinar cells extensively induced reprogramming of acinar cells into endocrine progenitor cells.

### Blood glucose control by the regenerated β cells

**Fig 6:**
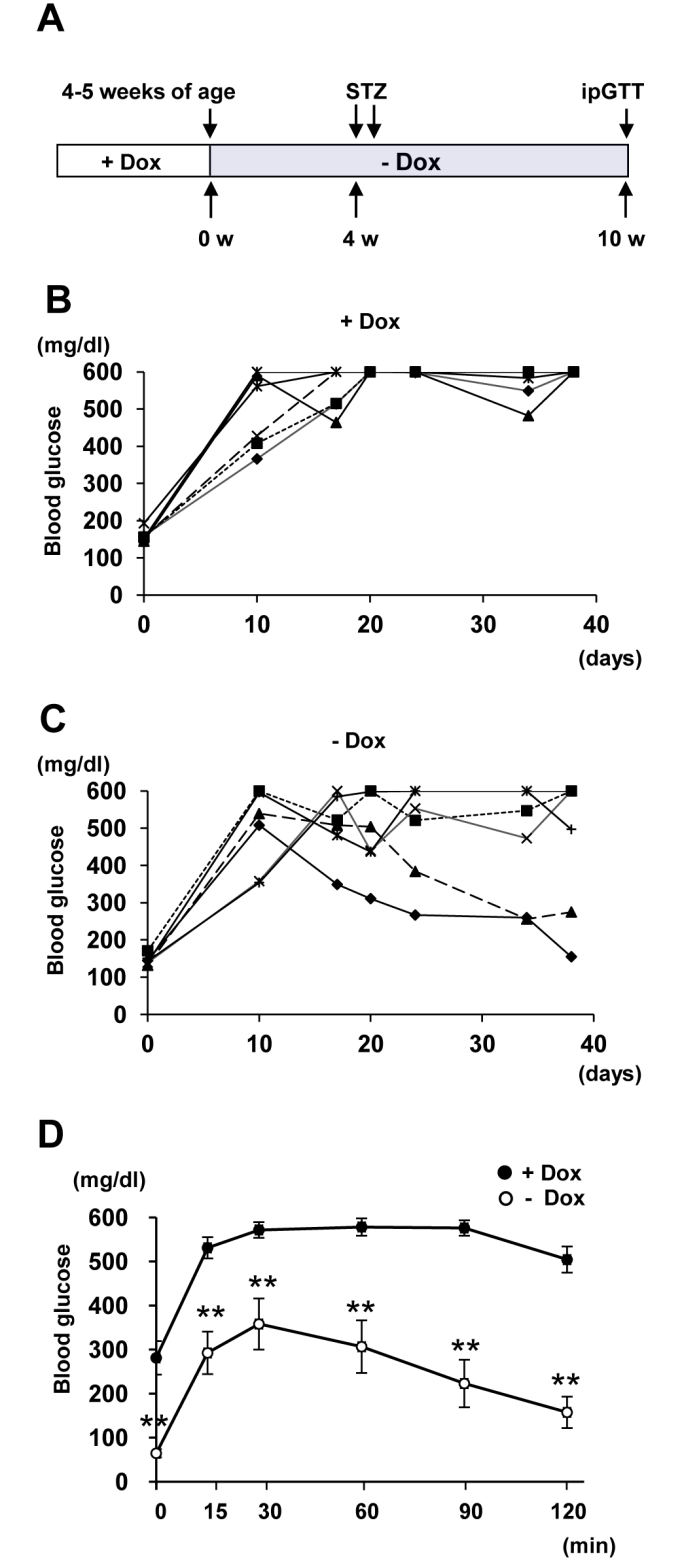
Effect of Pdx1 expression in ERTF-Pdx1-EGFP mice with STZ-induced diabetes. (A) Time course. ERTF-Pdx1-EGFP mice were given STZ 4 weeks after Dox withdrawal. Six weeks after STZ injection, the ipGTT was carried out and their pancreata were used for immunostaining analyses. (B-C) Non-fasting blood glucose levels were measured every three or ten days after STZ injection. Improvement of non-fasting blood glucose levels was seen in the ERTF-Pdx1-EGFP mice maintained without Dox (- Dox; *n* = 6). In contrast, sustained high blood glucose levels were seen in the Dox (+) ERTF-Pdx1-EGFP mice (+ Dox; *n* = 6). Note that the maximum glucose level of detection was 600 mg/dl. (D) The ipGTT was performed 6 weeks after STZ injection. The ERTF-Pdx1-EGFP mice maintained without Dox showed improved glucose tolerance (*n* = 6) compared with the Dox (+) ERTF-Pdx1-EGFP mice (*n* = 6). Data are shown as mean ± S.E. **P < 0.01 by Student’s *t*-test.

To further confirm that the EGFP-positive β cells observed in ERTF-Pdx1-EGFP mice after Dox withdrawal are newly generated, we used a mouse model of type 1 diabetes induced by STZ injection, which specifically causes islet β-cell death. The time course of the experiment was shown in Fig 6A. STZ was injected into double heterozygous ERTF-Pdx1-EGFP mice 4 weeks after Dox withdrawal, when few EGFP/insulin double-positive cells could be seen in the islets or islet-like clusters (Fig 4A and 4B). Without STZ treatment, ERTF-Pdx1-EGFP mice maintained without Dox for 10 to 11 weeks showed similar glucose tolerance compared with wild-type mice by an ipGTT (S6A Fig). If acinar cells were continuously reprogrammed into β cells, the newly generated β cells should gradually improve the glycemic control in the diabetic mice. Indeed, the STZ-induced hyperglycemia of the ERTF-Pdx1-EGFP mice maintained without Dox tended to improve over time, although their blood glucose levels were unstable (Fig 6C). In contrast, Dox (+) ERTF-Pdx1-EGFP control mice were overtly diabetic (Fig 6B).

Six weeks after the STZ injection, the mice were subjected to an ipGTT. Dox (+) ERTF-Pdx1-EGFP mice showed a typical diabetic response (Fig 6E, black circle), while the ERTF-Pdx1-EGFP mice maintained without Dox showed a much better glucose tolerance (Fig 6D, open circle). To verify that the improved glycemic control was owing to increased insulin release, we measured the serum insulin levels 30 min after the glucose injection (S6B Fig). The serum insulin levels of the STZ-treated ERTF-Pdx1-EGFP mice without Dox were significantly higher than those of the STZ-treated wild-type and Dox (+) ERTF-Pdx1-EGFP control mice, but lower than those of the untreated Dox (-) ERTF-Pdx1-EGFP and wild-type mice, probably because the number and/or function of the regenerated β cells was still below normal, consistent with the considerably reduced size of the islets in these animals (see below).

**Fig 7:**
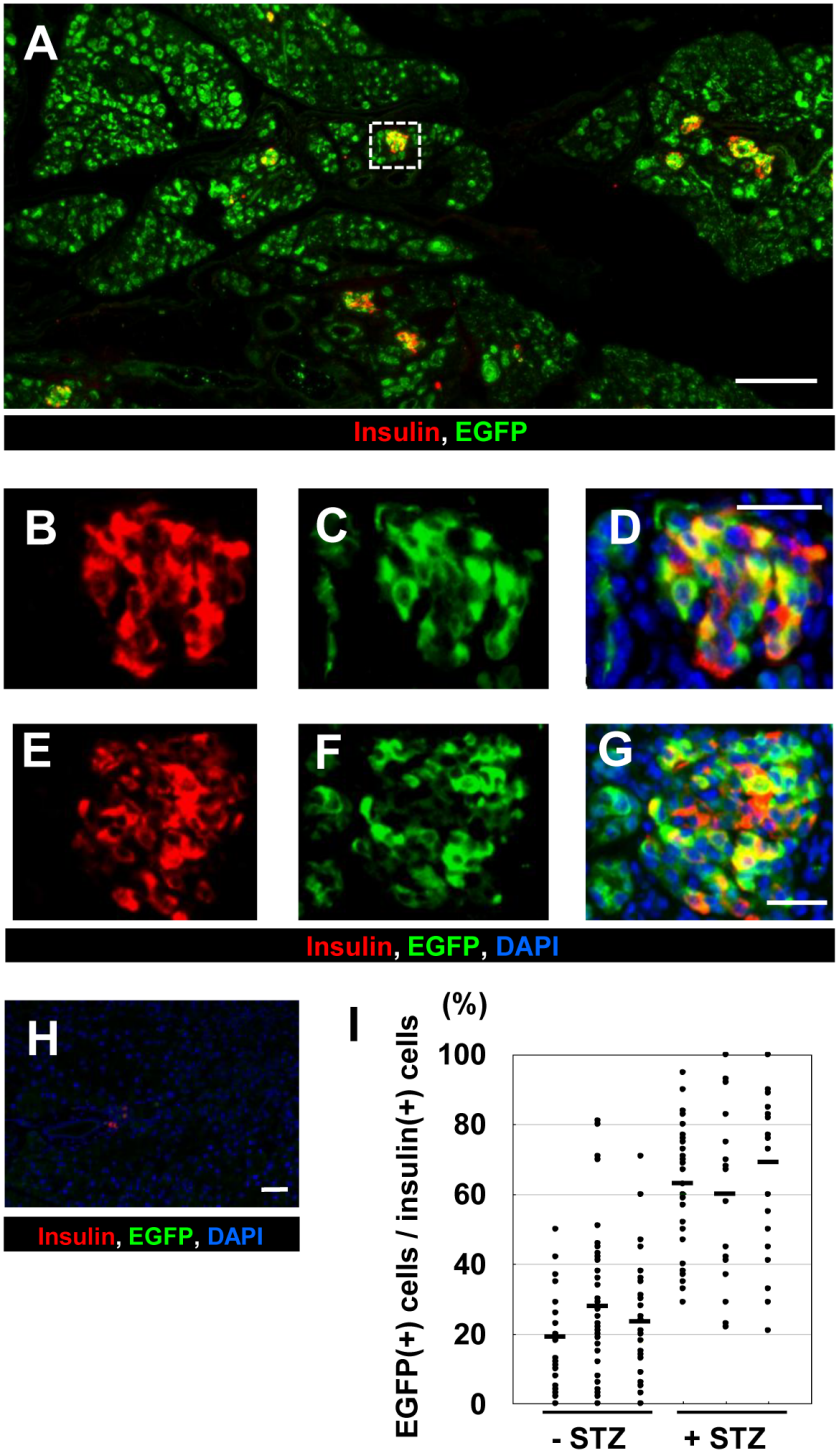
Immunohistochemical analysis of the pancreas of STZ-treated ERTF-Pdx1-EGFP mice without Dox. ERTF-Pdx1-EGFP mice were given STZ 4 weeks after Dox withdrawal. Six weeks after STZ injection, their pancreata were used for immunostaining analyses. (A) Immunostaining for insulin (red) and EGFP (green). Bar = 200 μm. (B-G) Magnified views of the islet regions including the dotted line-box in (A). Bars = 25 μm. Merged images including DAPI staining are also shown in (D) and (G). (H) Insulin-positive cells were rarely seen in the STZ-treated wild-type controls. (I) The percentage of EGFP-positive cells among insulin-positive islet cells was determined for each islet of the pancreas from three STZ-treated and three STZ-untreated ERTF-Pdx1-EGFP mice, 10 weeks after Dox withdrawal (*n* = 18–40 for each mouse). Each bar represents the mean for a single animal (19%, 28%, 24%, 63%, 60%, and 69%, respectively). The proportion of EGFP-positive cells among insulin-positive islet cells was significantly higher in the STZ-treated animals than in the STZ-untreated controls (*P* < 0.05).

We next examined pancreas sections from these mice with anti-GFP and anti-insulin staining (Fig 7A–7H). In the pancreas of STZ-treated Dox (+) ERTF-Pdx1-EGFP control mice, only a few islets including insulin-positive cells were found (Fig 7H). On the other hand, in the STZ-treated ERTF-Pdx1-EGFP mice 10 weeks after Dox withdrawal, the number of islets was not reduced compared with non-STZ-treated control mice, although their size was much smaller than in wild-type animals, because of the reduced number of β cells in each islet (Fig 7A). The percentage of EGFP-positive cells among the insulin-positive islet cells was nearly 100% in some islets and higher than 60% on average, much higher than in identical non-STZ-treated control mice (Fig 7I). Taken together, these results strongly support that the EGFP-positive β cells observed in ERTF-Pdx1-EGFP mice after Dox withdrawal are newly generated from Pdx1-expressing acinar cells and can function to control blood glucose levels *in vivo*.

## Discussion

Here we overexpressed Pdx1 in pancreatic acinar cells of the adult mouse and followed their fate using EGFP expression. These cells underwent acinar-to-ductal and/or acinar-to-β-cell transdifferentiation. Our control experiments showed that islet cells were unlikely to spontaneously become EGFP-positive. Our BrdU-labeling experiment showed that the EGFP/insulin double-positive islet cells were more proliferative than preexisting β cells (Fig 4I). To confirm that the EGFP/insulin double-positive cells seen in islets and islet-like clusters were newly generated, we treated ERTF-Pdx1-EGFP mice with STZ after Dox withdrawal. Forty days after STZ treatment, these mice showed much better glucose tolerance than controls in an ipGTT (Fig 6D), and the percentage of EGFP-positive cells among the insulin-positive cells (~60%; Fig 7I) was much higher than in equivalent STZ-untreated control mice (~25%), consistent with the hypothesis that the overexpressed Pdx1 induced β cell regeneration from acinar cells in these mice. However, if the STZ treatment almost completely ablated the β cells, as seen in the wild-type control mice, and if the exogenous Pdx1 expression induced β-cell regeneration in the STZ-injected mice, the EGFP/insulin double-positive cells should comprise nearly 100% of the insulin-positive islet cells. Our finding of ~60% EGFP/insulin double-positive cells seems lower than expected. We do not know the reason for this, but it may be because Pdx1-expressing acinar cells or acinar-derived cells gave some protective effects to preexisting islet β cells.

In addition to insulin-producing cells, we observed EGFP-positive somatostatin- and PP-producing cells which were probably derived from acinar cells, but not EGFP-positive glucagon-producing cells. This was probably because Pdx1 expression prevented the precursor cells from differentiating into α cells [46]. Interestingly, we also observed EGFP-positive islet cells that were not labeled with antibodies against insulin, somatostatin, PP, glucagon, PECAM-1, or HNF1β (Figs 5A–5E and 2R). Therefore, we favor the interpretation that Pdx1 expression induced the reprogramming of acinar cells into endocrine precursors that migrated into the islets and gradually differentiated into hormone-producing cells, although we do not have direct evidence for the precursor migration into the islets. Consistent with this interpretation, we observed endocrine-hormone-negative EGFP/Nkx6.1 double-positive islet cells in the pancreas of ERTF-EGFP mice 10 weeks after Dox withdrawal (Fig 5G–5J). Nkx6.1 is expressed in islet precursors and adult islet cells [43,44]. Recently, Schaffer et al. [47] showed that forced expression of Nkx6.1 in endocrine precursor cells of mice is sufficient to respecify non-β endocrine precursor cells towards the β cell lineage, while endocrine precursor- or β cell-specific inactivation of Nkx6.1 converts β cells to alternative endocrine lineages. Thus, these endocrine-hormone-negative EGFP/Nkx6.1 double-positive islet cells might be precursors towards the β cell lineage.

The neogenesis or budding of new islet cells from pancreatic ducts has been reported, although the existence and identity of the progenitor cells remains controversial. To test whether ductal cells serve as postnatal pancreatic progenitors, Inada et al. [48] genetically marked ductal cells with β-galactosidase using the Cre-lox system, with Cre driven by the duct-specific carbonic anhydrase II (CAII) promoter. They found β-galactosidase/insulin double-positive cells in islets in a pancreatitis model, with a distribution that was very similar to that of the EGFP/insulin double-positive cells described here. Xu et al. [32] showed that β-cell progenitors can be activated in the adult mouse pancreas following injury by duct ligation. These progenitors reside in the ductal lining, their differentiation is Ngn3-dependent, and they give rise to all islet cell types, including β cells. However, these events occurred only under the conditions of intense regenerative stimuli induced by duct ligation, which include a strong inflammatory response and massive loss of acinar cells.

In the context of these other studies, the transdifferentiation/reprogramming observed in our experiments may be explained as follows. Exogenous overexpression of Pdx1 inhibited the maintenance of the acinar cell phenotype [34] and caused their gradual loss, without inducing strong inflammation, leading to regenerative responses. Some of the remaining acinar cells dedifferentiated into pancreatic duct and/or precursor cells. The Pdx1 expression then induced the reprogramming of these cells into endocrine progenitor cells, which migrated into the islets and differentiated into insulin-, somatostatin-, or PP-producing endocrine cells. However, it is not clear if regenerative stimuli are essential for these steps, because EGFP-positive cells could be observed in islets 3 weeks after Dox withdrawal, when histological changes were not yet apparent in the exocrine pancreas. Alternatively, the Pdx1 expression might directly induce the reprogramming of acinar cells into insulin-producing cells without leading them into islets. The β cells in small, scattered clusters of less than ten cells are often interpreted as newly formed, precursor-derived β cells in the process of coalescing into larger or mature islets [49]. This model predicts that clusters newly formed after a chase should contain only EGFP-positive cells, which was, in fact, often the case in the Dox (-) ERTF-Pdx1-EGFP mice (Fig 4B–4E). Thus, the small clusters of EGFP-positive β cells might represent those directly derived from acinar cells.

Zhou et al. [50,51] reported that exocrine cells of adult mice could be reprogrammed to β-like cells *in vivo* by the AdV-mediated introduction of three transcription factor genes, Pdx1, Ngn3, and Mafa. In their experiments, the first β-like cells appeared as isolated cells or in small clusters 3 days after the AdV infection. By contrast, in our experiments, the reprogramming took much longer time. Their study may show the direct cellular reprogramming of acinar cells into β cells. Recently, Baeyens et al. [52] showed that transient administration of epidermal growth factor and ciliary neurotrophic factor to adult mice with chronic hyperglycemia efficiently stimulates the conversion of terminally differentiated acinar cells to β-like cells. This study also supported the plasticity of acinar cells. It would be interesting to examine the effects of the administration of these cytokines to the Dox (-) ERTF-Pdx1-EGFP mice.

In summary, our study shows that the forced expression of a single transcription factor, Pdx1, in pancreatic acinar cells is sufficient to induce their reprogramming into endocrine cells, including β cells. These newly generated β cells increased the serum insulin levels and ameliorated STZ-induced diabetes. These results are consistent with the idea that the forced expression of key transcription factors may be able to reverse terminal differentiation, to re-create a population of organ-specific precursor cells. The plasticity of the differentiated pancreatic cells, as evidenced by their reprogramming into *in vivo* progenitors for pancreatic endocrine cell types, suggests that reprogramming could be harnessed to treat diabetes by inducing the regeneration of new β cells.

## Supporting Information

S1 Fig Histological analysis of the pancreas of ERTF-Pdx1-EGFP mice. Hematoxylin and eosin staining of pancreas sections from RTFN-Pdx1-EGFP mice retaining the *loxP*-flanked neo^r^ gene (left panel) and from 2-week-old ERTF-Pdx1-EGFP mice that had never been treated with Dox (right panel). Bars = 50 μm.
(TIF)

S2 Fig Immunohistochemical analysis of control mouse pancreas. (A-D) Control mice carrying the Elastase-Cre transgene and the Tet-off knock-in cassette of RTFN-EGFP lacking the Pdx1 cDNA were maintained without Dox for 3, 6, and 9 weeks, and pancreas sections were stained for endocrine hormones (red), EGFP (green), and DNA (DAPI; blue). Bars = 200 μm. (E-J) Magnified views of the dotted line-boxes in (C). Staining of E-cadherin (light blue) was included in (F), (G), (I), and (J). There were no EGFP/insulin double-positive cells, demonstrating that there was no detectable spontaneous expression of Cre under the control of the Elastase promoter in the islets. Bars = 50 μm.
(TIF)

S3 Fig Immunohistochemical analysis for C-peptide. (A-D) Pancreas sections from ERTF-Pdx1-EGFP mice without Dox treatment for 3, 6, 9, and 16 weeks were stained for C-peptide (red), EGFP (green), and DNA (blue). The merged images are shown. The number of C-peptide/EGFP double-positive islet cells increased with time. (E) C-peptide-positive cells of control ERTF-Pdx1-EGFP mice (+ Dox) never co-expressed EGFP. Bar = 25 μm.
(TIF)

S4 Fig Immunohistochemical analysis for PP and somatostatin. (A-D) Immunohistochemical analysis of pancreas sections from ERTF-Pdx1-EGFP mice 3 and 6 weeks after Dox withdrawal. Staining for PP (red), EGFP (green), and DNA (blue) and their merged images (A) and (D) are shown. Arrows indicate EGFP/PP double-positive cells. Bars = 50 μm. (E-J) Immunohistochemical analysis of pancreas sections from ERTF-Pdx1-EGFP mice 9 and 10 weeks after Dox withdrawal. Staining for somatostatin (red), EGFP (green), E-cadherin (blue), and DNA (blue or light blue) and the merged images are shown. Magnified view of the dotted line-box in (E) is shown in (F). Magnified views of the dotted line-box in (G) are shown in (H) and (I). Arrows indicate EGFP/somatostatin double-positive cells. Arrowheads indicate an EGFP-negative somatostatin-positive cell. Bars = 50 μm.
(TIF)

S5 Fig Immunohistochemical analysis for MafB. (A-C) Immunohistochemical analysis of pancreas sections from wild-type mice (control). Staining for MafB (red), glucagon (light blue), and DNA (blue) and their merged image (C). Bars = 50 μm. (D-I) Immunohistochemical analysis of pancreas sections from ERTF-Pdx1-EGFP mice 10 weeks after Dox withdrawal. Staining for EGFP (green), MafB (red), DNA (blue), and endocrine hormones (light blue) and their merged images (H) and (I) are shown. Arrow indicates an islet. Arrowhead indicates EGFP/MafB double-positive endocrine hormone-negative cells. EGFP-negative MafB-positive endocrine hormone-positive islet cells are α cells. Bars = 50 μm. (J-Q) Staining for MafB (green), Amylase (red), Hnf1β (light blue), and DNA (blue) and their merged images (N-Q) are shown. Arrows indicate MafB-positive Hnf1β/amylase negative cells. Bars = 50 μm.
(TIF)

S6 Fig Glucose tolerance test and serum insulin levels. (A) The ipGTT was performed in STZ-untreated controls. Black circles represent the result of STZ-untreated wild-type controls (*n* = 5) and open circles represent that of STZ-untreated ERTF-Pdx1-EGFP mice maintained without Dox (*n* = 3). Data are shown as mean ± S.E. *P < 0.05 by Student’s *t*-test. (B) The experimental groups were STZ-treated wild-type mice (*n* = 4), STZ-treated Dox (+) ERTF-Pdx1-EGFP mice (*n* = 4), STZ-treated Dox (-) ERTF-Pdx1-EGFP mice (*n* = 4), untreated Dox (-) ERTF-Pdx1-EGFP mice (*n* = 5), and untreated wild-type mice (*n* = 8). Blood samples were obtained 30 min after glucose injection, and the insulin concentrations were measured using an ELISA kit. Statistical analyses were carried out by Student’s *t* test or by one-way ANOVA followed by Tukey’s post-hoc test for comparison among three STZ-treated groups. *P < 0.05, **P < 0.01.
(TIF)
