# Supplementary figures and images for: Transgenic Expression of a Single Transcription Factor Pdx1 Induces Transdifferentiation of Pancreatic Acinar Cells to Endocrine Cells in Adult Mice
Source: PLoS One. 2016 Aug 15;11(8):e0161190. doi: 10.1371/journal.pone.0161190 (PMC4985130; doi:10.1371/journal.pone.0161190)

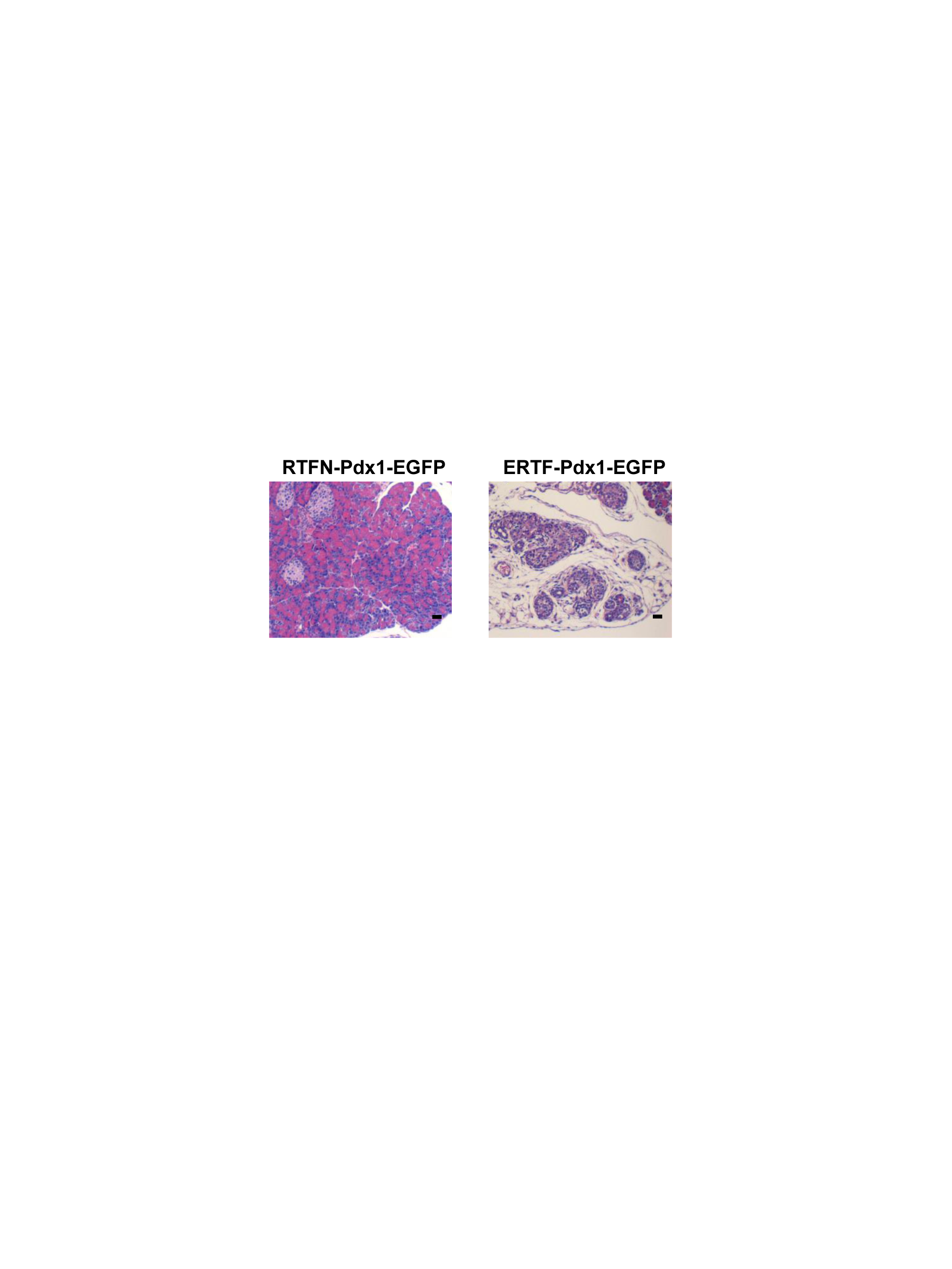

Supplement: S1 Fig — Hematoxylin and eosin staining of pancreas sections from RTFN-Pdx1-EGFP mice retaining the loxP-flanked neor gene (left panel) and from 2-week-old ERTF-Pdx1-EGFP mice that had never been treated with Dox (right panel). Bars = 50 μm. (TIF) [file pone.0161190.s001.tif]

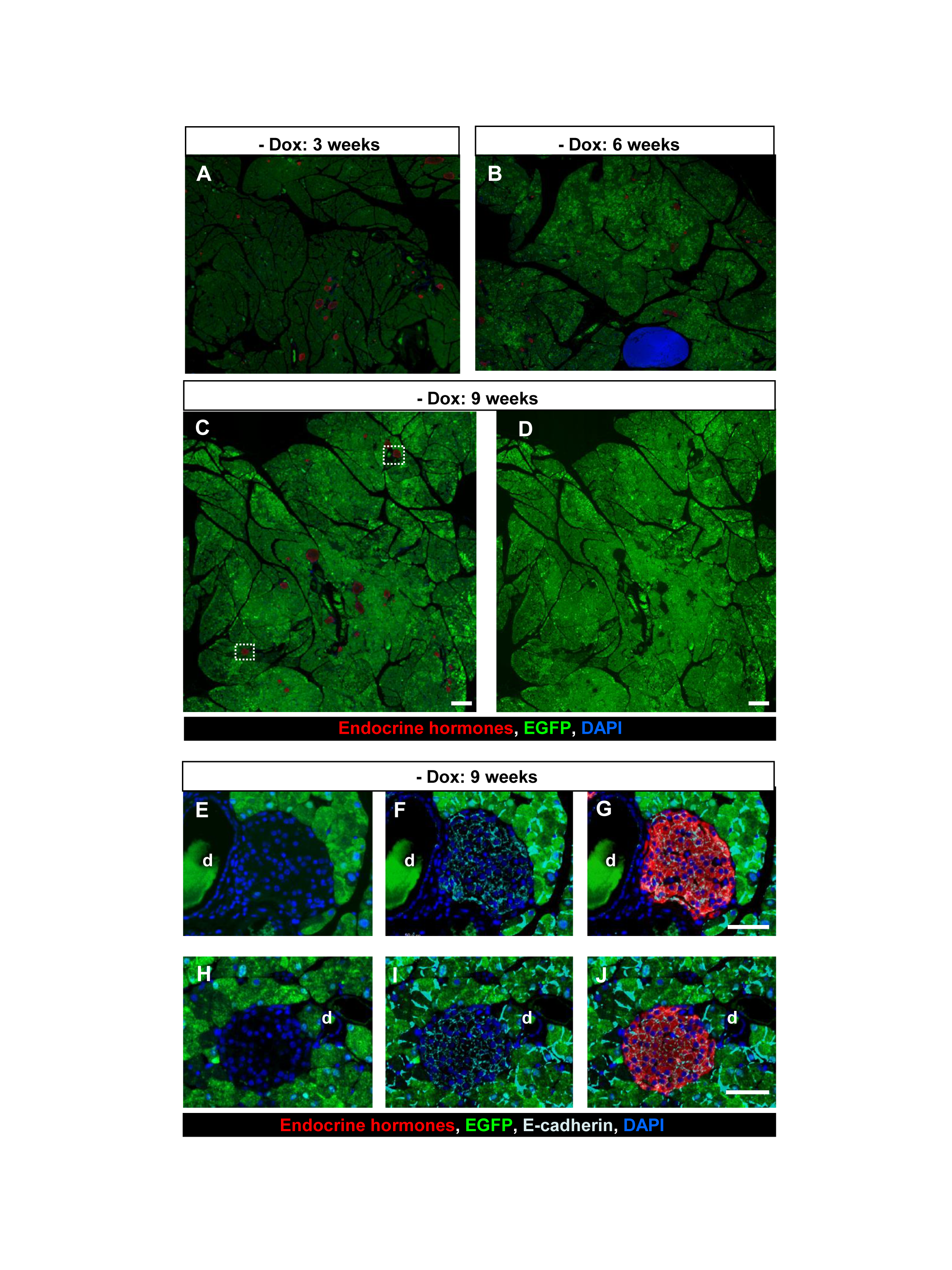

Supplement: S2 Fig — (A-D) Control mice carrying the Elastase-Cre transgene and the Tet-off knock-in cassette of RTFN-EGFP lacking the Pdx1 cDNA were maintained without Dox for 3, 6, and 9 weeks, and pancreas sections were stained for endocrine hormones (red), EGFP (green), and DNA (DAPI; blue). Bars = 200 μm. (E-J) Magnified views of the dotted line-boxes in (C). Staining of E-cadherin (light blue) was included in (F), (G), (I), and (J). There were no EGFP/insulin double-positive cells, demonstrating that there was no detectable spontaneous expression of Cre under the control of the Elastase promoter in the islets. Bars = 50 μm. (TIF) [file pone.0161190.s002.tif]

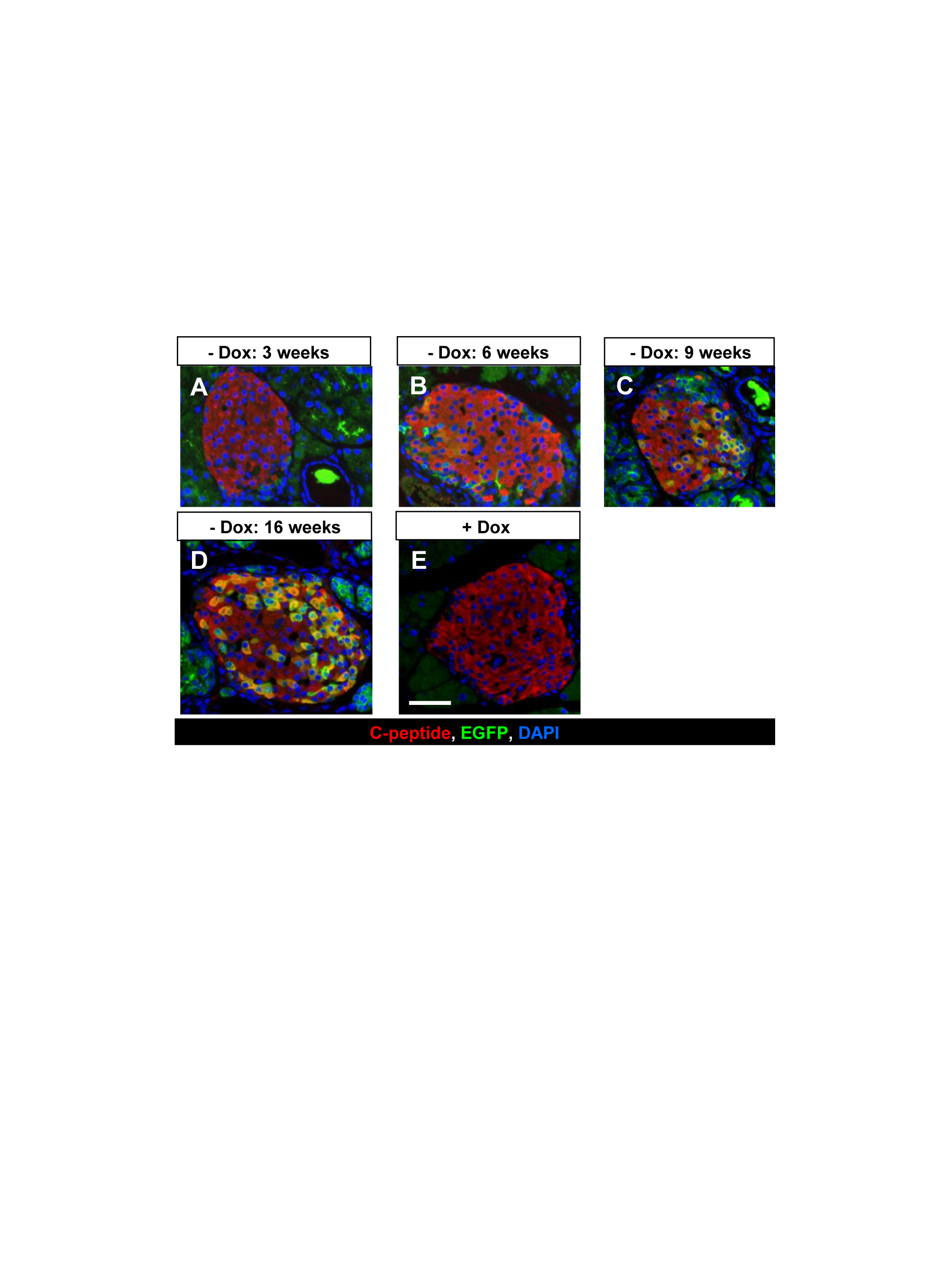

Supplement: S3 Fig — (A-D) Pancreas sections from ERTF-Pdx1-EGFP mice without Dox treatment for 3, 6, 9, and 16 weeks were stained for C-peptide (red), EGFP (green), and DNA (blue). The merged images are shown. The number of C-peptide/EGFP double-positive islet cells increased with time. (E) C-peptide-positive cells of control ERTF-Pdx1-EGFP mice (+ Dox) never co-expressed EGFP. Bar = 25 μm. (TIF) [file pone.0161190.s003.tif]

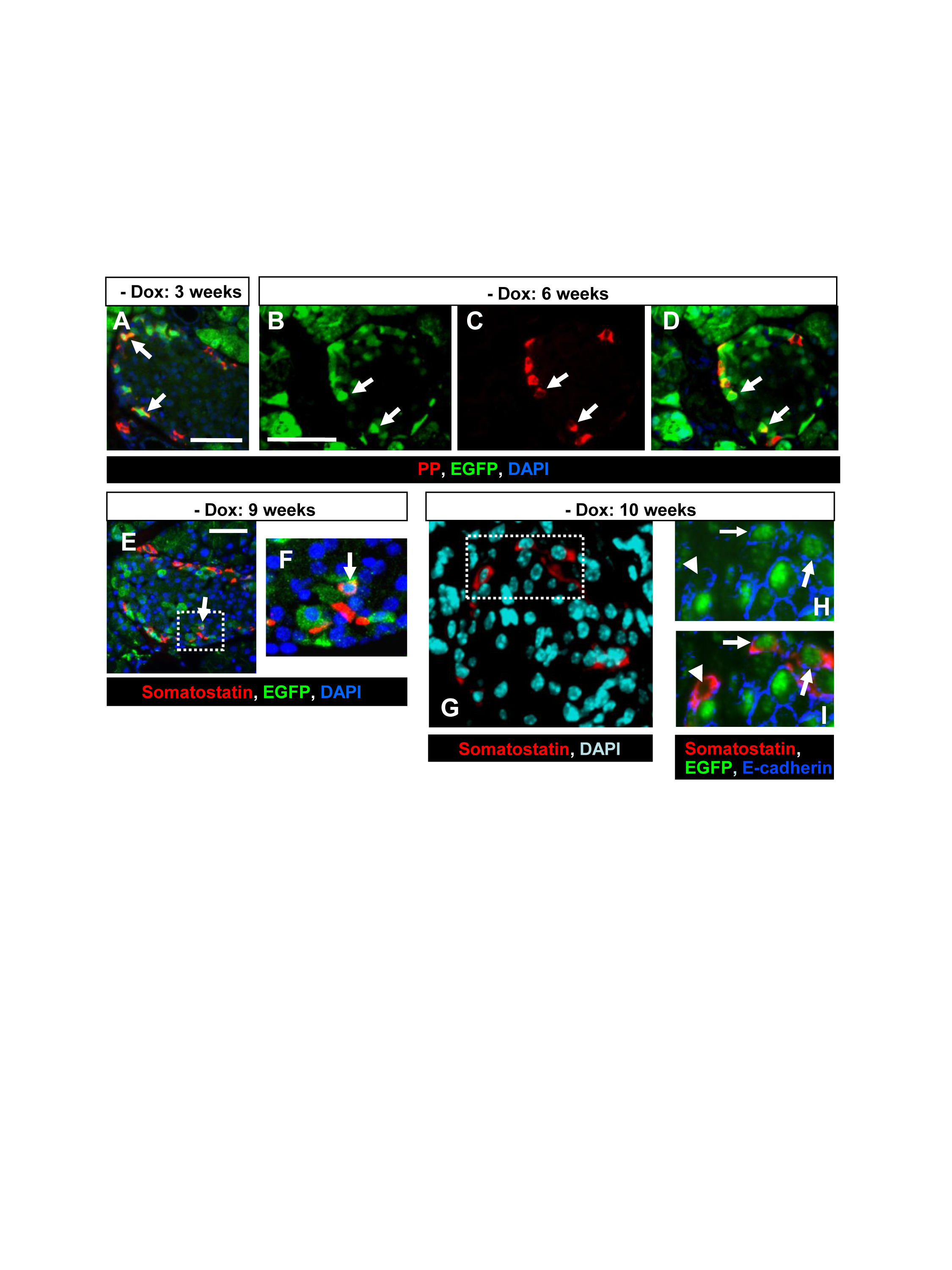

Supplement: S4 Fig — (A-D) Immunohistochemical analysis of pancreas sections from ERTF-Pdx1-EGFP mice 3 and 6 weeks after Dox withdrawal. Staining for PP (red), EGFP (green), and DNA (blue) and their merged images (A) and (D) are shown. Arrows indicate EGFP/PP double-positive cells. Bars = 50 μm. (E-J) Immunohistochemical analysis of pancreas sections from ERTF-Pdx1-EGFP mice 9 and 10 weeks after Dox withdrawal. Staining for somatostatin (red), EGFP (green), E-cadherin (blue), and DNA (blue or light blue) and the merged images are shown. Magnified view of the dotted line-box in (E) is shown in (F). Magnified views of the dotted line-box in (G) are shown in (H) and (I). Arrows indicate EGFP/somatostatin double-positive cells. Arrowheads indicate an EGFP-negative somatostatin-positive cell. Bars = 50 μm. (TIF) [file pone.0161190.s004.tif]

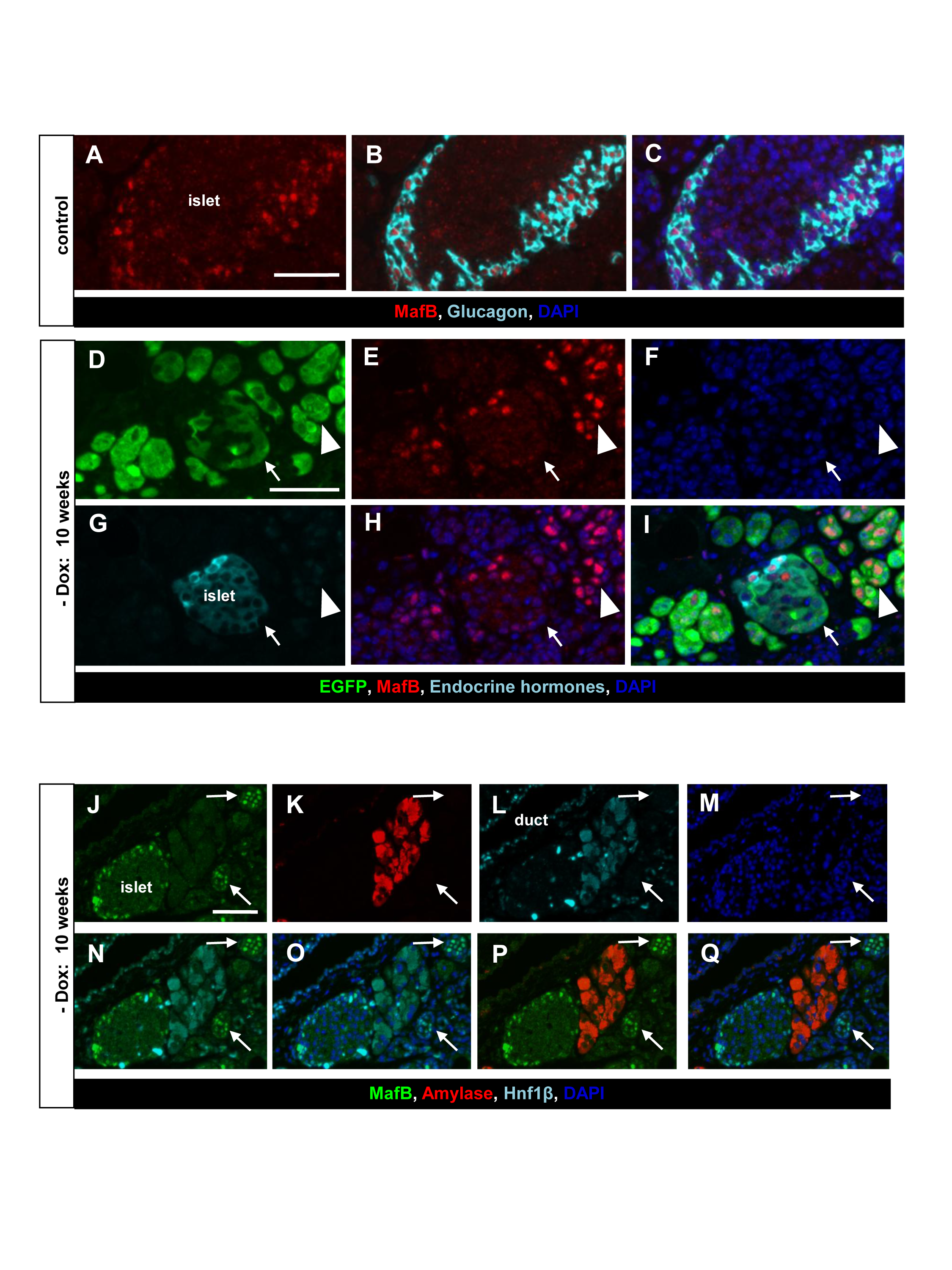

Supplement: S5 Fig — (A-C) Immunohistochemical analysis of pancreas sections from wild-type mice (control). Staining for MafB (red), glucagon (light blue), and DNA (blue) and their merged image (C). Bars = 50 μm. (D-I) Immunohistochemical analysis of pancreas sections from ERTF-Pdx1-EGFP mice 10 weeks after Dox withdrawal. Staining for EGFP (green), MafB (red), DNA (blue), and endocrine hormones (light blue) and their merged images (H) and (I) are shown. Arrow indicates an islet. Arrowhead indicates EGFP/MafB double-positive endocrine hormone-negative cells. EGFP-negative MafB-positive endocrine hormone-positive islet cells are α cells. Bars = 50 μm. (J-Q) Staining for MafB (green), Amylase (red), Hnf1β (light blue), and DNA (blue) and their merged images (N-Q) are shown. Arrows indicate MafB-positive Hnf1β/amylase negative cells. Bars = 50 μm. (TIF) [file pone.0161190.s005.tif]

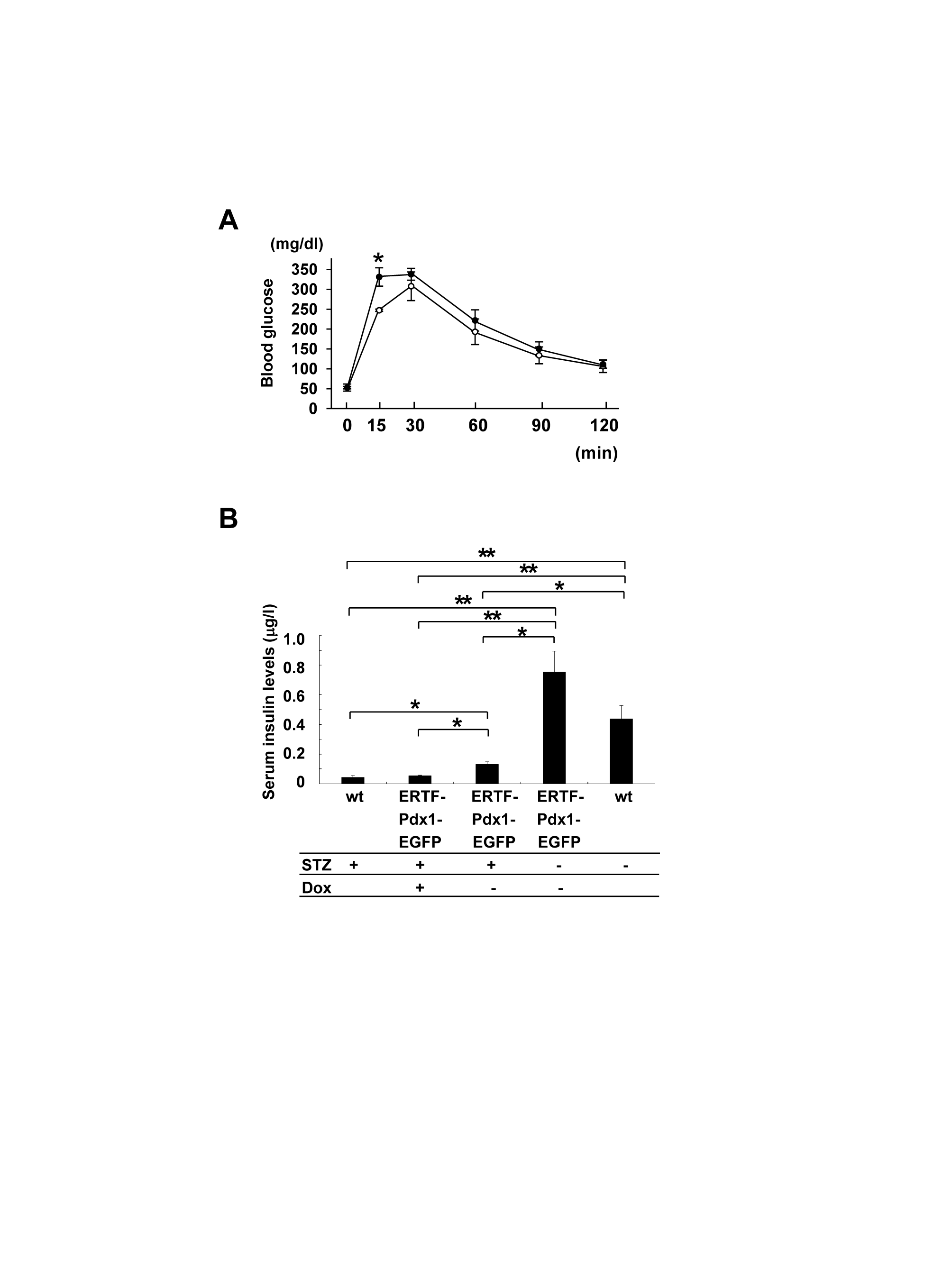

Supplement: S6 Fig — (A) The ipGTT was performed in STZ-untreated controls. Black circles represent the result of STZ-untreated wild-type controls (n = 5) and open circles represent that of STZ-untreated ERTF-Pdx1-EGFP mice maintained without Dox (n = 3). Data are shown as mean ± S.E. *P < 0.05 by Student’s t-test. (B) The experimental groups were STZ-treated wild-type mice (n = 4), STZ-treated Dox (+) ERTF-Pdx1-EGFP mice (n = 4), STZ-treated Dox (-) ERTF-Pdx1-EGFP mice (n = 4), untreated Dox (-) ERTF-Pdx1-EGFP mice (n = 5), and untreated wild-type mice (n = 8). Blood samples were obtained 30 min after glucose injection, and the insulin concentrations were measured using an ELISA kit. Statistical analyses were carried out by Student’s t test or by one-way ANOVA followed by Tukey’s post-hoc test for comparison among three STZ-treated groups. *P < 0.05, **P < 0.01. (TIF) [file pone.0161190.s006.tif]
